# Wave Function Engineering on Superconducting Substrates: Chiral Yu-Shiba-Rusinov Molecules

**DOI:** 10.1021/acsnano.4c10998

**Published:** 2024-10-25

**Authors:** Lisa M. Rütten, Harald Schmid, Eva Liebhaber, Giada Franceschi, Ali Yazdani, Gaël Reecht, Kai Rossnagel, Felix von Oppen, Katharina J. Franke

**Affiliations:** † Fachbereich Physik, Freie Universität Berlin, 14195 Berlin, Germany; ‡ Dahlem Center for Complex Quantum Systems and Fachbereich Physik, Freie Universität Berlin, 14195 Berlin, Germany; § Institut für Experimentelle und Angewandte Physik, Christian-Albrechts-Universität zu Kiel, 24098 Kiel, Germany; ∥ Ruprecht Haensel Laboratory, Deutsches Elektronen-Synchrotron DESY, 22607 Hamburg, Germany

**Keywords:** Yu-Shiba-Rusinov states, chirality, superconductivity, niobium diselenide, scanning tunneling microscopy

## Abstract

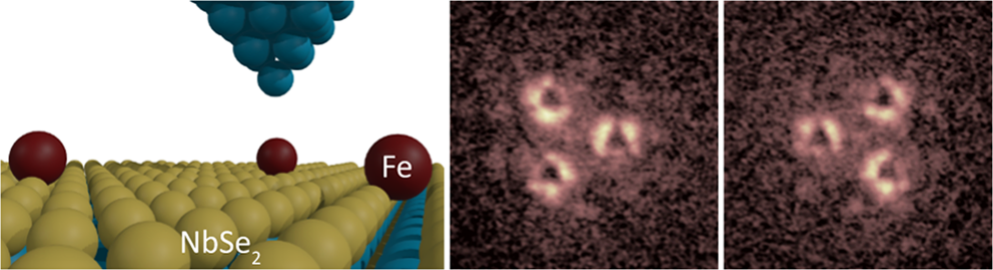

Magnetic adatoms on superconductors give rise to Yu-Shiba-Rusinov (YSR) states that hold considerable interest for the design of topological superconductivity. Here, we show that YSR states are also an ideal platform to engineer structures with intricate wave function symmetries. We assemble structures of iron atoms on the quasi-two-dimensional superconductor 2*H*-NbSe_2_. The Yu-Shiba-Rusinov wave functions of individual atoms extend over several nanometers enabling hybridization even at large adatom spacing. We show that the substrate can be exploited to deliberately break symmetries of the adatom structure leading to hybridized YSR states exhibiting symmetries that cannot be found in orbitals of iso-structural planar molecules in the gas phase. We exploit this potential by designing chiral YSR wave functions of triangular adatom structures. Our results significantly expand the range of interesting quantum states that can be engineered using arrays of magnetic adatoms on superconductors.

Structures of magnetic adsorbates on superconductors have recently garnered significant attention, primarily due to their pivotal role in the pursuit of topological superconductivity.^1^ The building blocks are Yu-Shiba-Rusinov (YSR) states, which arise from the exchange interaction of magnetic adatoms with the conduction electrons of the underlying superconductor.^2−5^ In his seminal work, Rusinov predicted that YSR states originating from two nearby impurities can hybridize forming symmetric and antisymmetric combinations of the YSR wave functions.^4^ This phenomenon of hybridization has been experimentally observed in both self-assembled and artificially constructed dimers.^6−10^ Extended structures such as YSR chains and lattices exhibit YSR bands and have been investigated as promising platforms for topological superconductivity.^11−19^

More generally, artificial lattices on surfaces hold great potential for the design of two-dimensional structures that feature lattice geometries with desired properties, such as flat bands,^20^ topological insulator states,^21^ or fractality.^22^ Common to these realizations is that a metal substrate introduces significant broadening of the relevant states. Attempts to create adatom structures decoupled from the bulk included atomic arrangements on the surface of a semiconductor. There, a two-dimensional electron gas at the surface mediates efficient coupling between the adatoms while at the same time isolating them from the bulk bands.^23^ Superconducting substrates are advantageous in that they decouple adatom states even more efficiently if located inside the superconducting gap, which is the case for YSR states. The use of a quasi-two-dimensional superconductor such as 2*H*-NbSe_2_ is beneficial, since it supports YSR states that are long-ranged parallel to the surface,^24^ but decay rapidly into the bulk.

Beyond providing electronic states that communicate a coupling among adatoms, the crystalline substrate introduces an additional interesting feature: The local symmetry of an adsorption site governs the crystal-field splitting of the states. Singly occupied states exchange couple to the substrate and induce long-ranged YSR states on a quasi-two-dimensional superconductor. The symmetry of these states is given by the local exchange scattering potential and the Fermi surface. The resulting YSR states can be used as building blocks arranged in variable configurations on the substrate for designing larger structures. Thus, the symmetry of the substrate adds interesting ways to realize artificial molecules embedded in nontrivial environments, opening alternative opportunities for wave function design.

Here, we use a scanning tunneling microscope (STM) to build and investigate structures of iron (Fe) atoms built atom by atom on a 2*H*-NbSe_2_ crystal. Surprisingly, we find that the spectra recorded on top of the two atoms forming a dimer differ from each other when the dimer lacks an inversion center or a perpendicular mirror plane. Such distinct spectra on individual atoms are not typically encountered in hybridized YSR dimers, emphasizing the significant influence of crystal symmetry and adsorption geometry on adatom assemblies. Employing model calculations of corresponding YSR assemblies on NbSe_2_, we rationalize the experimental shapes and their symmetries. We exploit the symmetries of the YSR dimer to build larger two-dimensional structures and realize a versatile platform for YSR wave function engineering as exemplified by the realization of chiral YSR molecules.

## Results and Discussion

### YSR Wave Functions of Fe Monomers and Dimers

**Figure 1:**
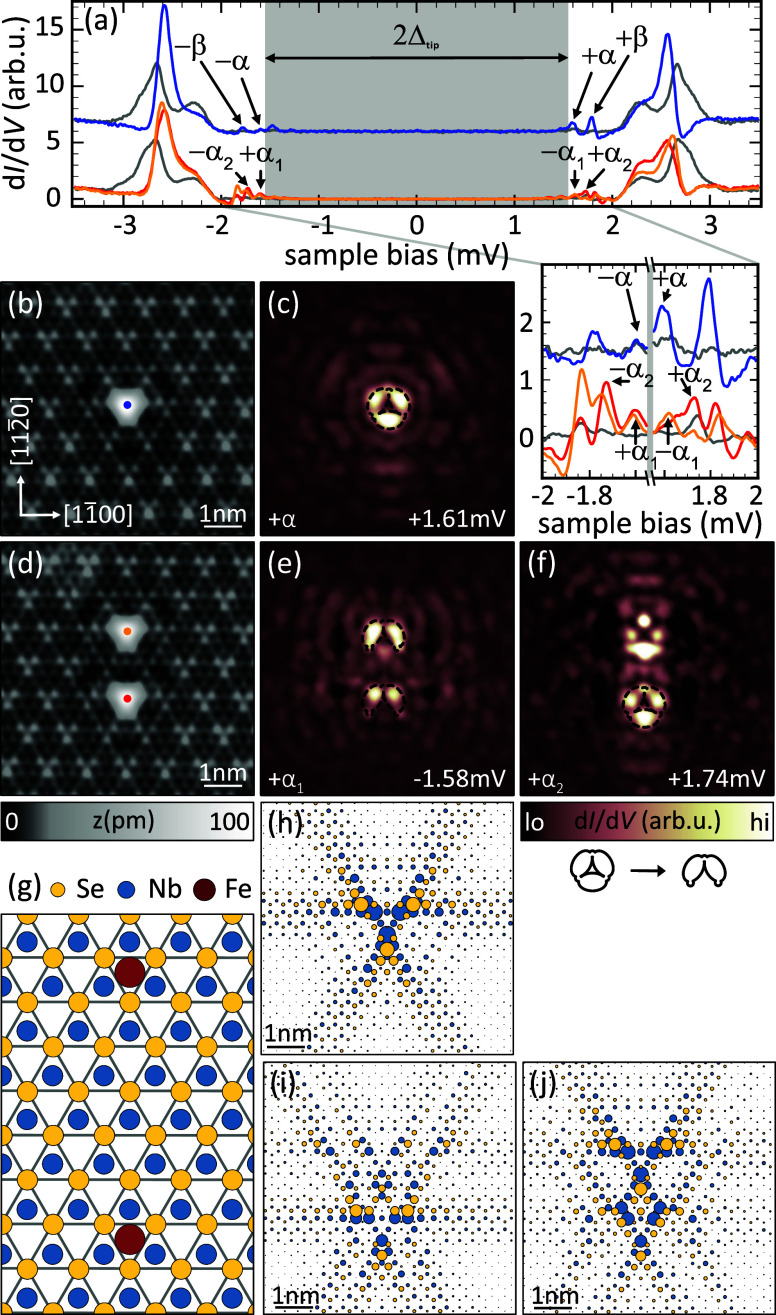
Fe monomer and dimer on the 2*H*-NbSe_2_ surface. (a) Tunneling spectra recorded on the Fe monomer and dimer adsorbed at CDW maxima of the NbSe_2_ substrate. For the color coding, refer to panels (b, d). Spectra of the NbSe_2_ substrate are shown in gray. (b), (d) Atomic resolution images of the monomer and dimer. (c, e, f) Corresponding constant-contour dI/dV maps of the +α-resonance (monomer) and +α_1,2_-resonances (dimer) with area and scale as in (b, d). The reduction of the characteristic +α shape is depicted below the color bar of (f). (g) Adsorption geometry of Fe adatoms (red circles) in the dimer configuration. (h) Numerical tight-binding calculation of the YSR monomer state (electron-like component). The lattice sites are color coded as in (g) (blue for Nb and yellow for Se) and the diameter of the circles indicates the magnitude of the local density of states of the YSR state at the respective location. For parameters see Supporting Information (SI) Note 3. (i, j) Symmetric and antisymmetric dimer wave functions. The adsorption sites are the same as in the experiment. Δ_tip_ = 1.55 mV; set points: (b), (d) 10 mV, 100 pA; (c), top row of (a) 5 mV, 250 pA; bottom row of (a, e, f) 5 mV, 700 pA; all: *V*_rms_ = 15 μV.

When constructing structures from Fe atoms on NbSe_2_, one has to consider the effects of the charge density wave (CDW) that coexists with superconductivity in NbSe_2_ at low temperatures. The YSR energy and the spatial extent and symmetry of the YSR wave functions are strongly influenced by the charge-density modulations.^25^ To ensure that all atoms within our structures would individually exhibit equivalent spectra, we position all atoms in hollow sites of the crystal that coincide with maxima of the CDW. Figure 1b shows a topography of an Fe atom sitting in a hollow site at a CDW maximum (the position with respect to the lattice is indicated in Figure 1g). The corresponding spectrum is depicted in blue in Figure 1a with a spectrum of the bare substrate shown in gray for comparison. We observe four YSR states, two of which lie within the range of the substrates coherence peaks originating from the highly anisotropic band structure of NbSe_2_.^26^ Here, we focus on the YSR state deepest in the superconducting energy gap owing to its sharpness and characteristic spatial shape (labeled α in Figure 1a).

Figure 1c shows a differential conductance (d*I*/d*V*) map recorded at the energy of the +α resonance (+referring to its observation at positive bias voltage). It exhibits a distinct shape consisting of three lobes of high intensity arranged in a triangle around the atom’s center. This shape was also found in previous experiments.^16,25,27^ A sketch of the shape is overlaid as a guide to the eye in Figure 1c. Further from the atom’s center, an oscillating, 6-fold pattern is observed. Generally, YSR wave functions inherit their short-range characteristics from the *d*-level hosting the unpaired electron spin that gives rise to the YSR state.^28^ The *d*-levels in turn are subject to a crystal field when the atom is adsorbed on a surface and therefore inherit the 3-fold symmetry of the adsorption geometry. Here, all atoms are adsorbed in high-symmetry positions of the substrate both with respect to the lattice and the CDW. Correspondingly, we observe *D*_3_ symmetry (3-fold rotational symmetry including mirror axes) in both the topography (Figure 1b) and the d*I*/d*V* map (Figure 1c). The long-range oscillations of the YSR wave function reflect the hexagonal Fermi surface of the substrate with the periodicity given by the Fermi wave vector *k*_F_.^24,28^

We form dimers by positioning a second Fe atom at a next-nearest CDW maximum. This maximum is located at a distance of ≈1.8 nm along the [112̅0] direction, i.e., perpendicular to an atomic row, and corresponding to a spacing of 3√3*a* (with *a* being the lattice constant). The schematic in Figure 1g depicts the positions of the Fe atoms on the NbSe_2_ surface. A topographic image of the dimer is shown in Figure 1d. Spectra recorded on both dimer atoms exhibit an increased number of YSR resonances compared to the monomer (most clearly seen in the close-up view of Figure 1a). This larger number of resonances is a first hint toward hybridization of the YSR wave functions leading to the formation of a YSR molecule. We assign the lowest two resonances as split α resonances (named as α_1_ and α_2_).

The assignment is corroborated by the d*I*/d*V* maps of the lowest-energy resonances (see Supporting Information (SI) Note 6 for more details). These maps show signatures of the characteristic pattern of the monomer’s +α resonance as indicated by the overlaid sketches in Figure 1e,f. In contrast to earlier observations on Fe dimers oriented along the [11̅00] direction (along the close packed Se rows), the resonances exhibit different intensities on the two atoms (compare red and orange spectra in Figure 1a), which is also reflected in the d*I*/d*V* maps recorded on the dimer. Here, the *D*_3_ symmetry is reduced to a single mirror axis. This symmetry reduction is highlighted by the change in the characteristic +α shape as depicted below the color bars in Figure 1f. We note that one of the +α derived resonances, namely the +α_1_ resonance, has shifted to negative bias. This transition through zero energy can be attributed to a quantum phase transition where an originally screened impurity spin with a bound quasi-particle becomes unscreened upon dimer formation. The phase transition is driven by the energy gain in Ruderman–Kittel–Kasuya–Yosida (RKKY) coupling,^29−31^ similar to the observation in differently arranged Fe dimers on 2*H*-NbSe_2_.^16^ Furthermore, the map at higher energy (Figure 1f) exhibits characteristics of the +β resonance alongside those of the +α resonance, indicating an energetic overlap of the two hybridized states (for more details see SI Note 6). Unlike d*I*/d*V* maps of hybridized YSR states observed in previous experiments, neither of our +α maps exhibits a nodal plane between the atoms. The observation of a nodal plane for the antisymmetric hybrid state is widely considered a clear feature of hybridization.^6,16,29,32,33^ In the following, we show how this apparent contradiction is resolved.

The reduced symmetry in the YSR states of the dimer can be interpreted by simulating the spatial structure of YSR states of single atoms and assembling dimers according to the experimental geometry. We model the NbSe_2_ substrate by an effective tight-binding description of Nb and Se orbitals on hexagonal lattices. The Fe adatoms are placed in hollow adsorption sites of the NbSe_2_ lattice and treated as a classical spin impurity with isotropic exchange and potential scattering to both Nb and Se neighbors. As the Fe monomer shows long-range 6-fold oscillations of wavelength λ_F_ = 1 nm, compatible with the Fermi momentum *k*_F_ = 5.34 nm^–1^ of the *K*-pockets, we restrict the coupling to the *K*-pockets of the Fermi surface.^34^ Different from previous models with dominant coupling to the Nb sites,^24^ we also include coupling to the Se sites. The simulations yield a pattern that qualitatively resembles the experimental *D*_3_ symmetry in the YSR pattern close to the Fe atom and exhibits a 6-fold symmetry in the far field (Figure 1h). We note that our simulations do not account for the *d*-orbital structure of the adatoms, which precludes a more quantitative comparison. For details, see SI Note 1.

Extending the same approach to adatom dimers, we obtain the two YSR wave functions for the experimental dimer configuration as depicted in Figure 1i,j. We observe that there is no mirror plane perpendicular to the dimer axis and the intensity distribution around the adatoms of the dimer is asymmetric. As for the monomer, the model thus correctly reproduces the symmetries observed in experiment well. In particular, our simulations are in agreement with the absence of a mirror plane perpendicular to the dimer axis.

The asymmetry can also be understood within a simpler phenomenological model, which considers the YSR pattern of a single Fe atom to be a linear superposition of *s*-wave YSR states centered at the positions of the Nb atoms, i.e., at an equilateral triangle around the Fe atom (Figure 1c, see SI Notes 2, 3). This approach is motivated by the 3-fold symmetry of the adatom environment so that the effective dominant exchange coupling is to neighboring Nb atoms. The dimer wave functions can then be simulated as symmetric and antisymmetric linear combinations of the monomer states. This simple model captures the main symmetries found in experiment very well.

To further test our tight-binding and phenomenological models, we also simulated YSR wave functions of dimers with a mirror plane between both atoms (i.e., along the [11̅00] direction at a spacing of three lattice sites ≈1 nm). Such dimers were probed and discussed in the context of dilute YSR chains in ref (16). The models correctly reproduce the experimentally found symmetries as shown in the SI Notes 3 and 6. The success of both models implies that one can readily design the symmetries of YSR wave functions in coupled adatom structures by exploiting the adsorption sites of the individual atoms and their positioning relative to each other and the underlying substrate.

### Chiral YSR Wave Functions of Fe Trimers

**Figure 2:**
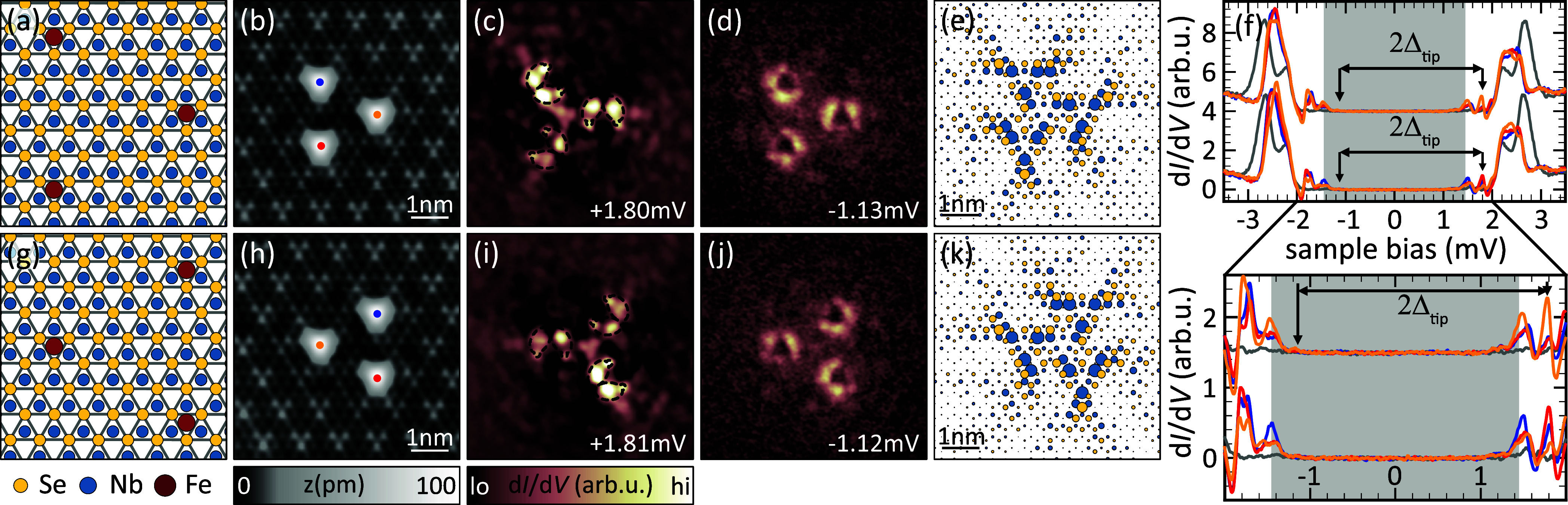
Chiral Fe triangles on NbSe_2_. (a, g) Schematic adsorption geometries of chiral Fe-trimer structures. (b, h) Topographic images of differently oriented Fe triangles. (c, i) Exemplary d*I*/d*V* map of a +α resonance for each enantiomer. (d, j) d*I*/d*V* maps of the thermal replica of the resonances mapped in (c, i) (intensities multiplied by a factor of 10 to compensate for the reduced intensity of thermal replica). (f) Spectra recorded on each of the atoms in the triangles depicted in (b) (top row) and (h) (bottom row). The arrows indicate the energies at which the maps were recorded. (e, k) One YSR state of each enantiomer as obtained from our tight-binding model. Δ_tip_ = 1.44 mV; set points: (b, h) 10 mV; 50 pA; rest 5 mV, 750 pA; all: *V*_rms_ = 15 μV.

We can further exploit the potential of reduced symmetry by constructing larger adatom structures in the experiment. We begin by arranging the atoms into an equilateral triangle by adding a third atom to the dimer (see Figure 2a for a schematic of the adsorption geometry). The third atom breaks the vertical mirror symmetry of the dimer, leaving only rotational (*C*_3_) symmetry.

To probe the effect of broken symmetry on the YSR states, we first need to confirm the presence of hybridized YSR states. The corresponding d*I*/d*V* spectra on the individual atoms are shown in Figure 2f. It is difficult to resolve well-separated resonances in these spectra. Yet, there is a clear change from the spectra of the monomer or dimer with an increased number of peaks. This increased number of resonances suggests further hybridization albeit with partial energetic overlap of the hybridization-derived resonances within our energy resolution. This experimental limitation may not be surprising considering the expectation of three hybrid states stemming from each of the four YSR resonances within the superconducting energy gap of only about 1 meV. Figure 2c shows the d*I*/d*V* map at the energy of the sharpest peak among those derived from the α resonance of the monomer (for the identification see below). Interestingly, this pattern reveals a clear handedness, exhibiting only a 3-fold rotational symmetry (*C*_3_) without any mirror plane. Such structures are typically referred to as chiral and we thus refer to this structure as a chiral YSR molecule. We identify characteristics of the +α derived state, which we highlight by the overlaid (reduced) characteristics. The black regions in the d*I*/d*V* map correspond to negative differential conductance, that occurs because we probe the energetically sharp YSR resonances with the sharp coherence peaks of the superconducting tip.

To disentangle the actual contributions of a resonance from negative differential conductance of a close-by resonance, we look at the thermal replica of the resonance of interest. Energetically, these thermal replica are located within the energy gap of the tip (here indicated by the gray area in spectra) and shifted across zero bias by twice the superconducting gap of the tip (see SI Note 5 for details). A d*I*/d*V* map of the thermal replica of the resonance depicted in Figure 2c is shown in Figure 2d. The energies at which both maps were recorded are indicated by arrows in the spectra in Figure 2f. The +α-like shape as well as the handedness are more clearly visible in Figure 2d, but the general features are not drastically changed. Figure 2h–j shows an equivalent data set for another trimer arrangement, i.e., where the third atom is added on the other side of the dimer (schematic of adsorption geometry in Figure 2g). This change in configuration should produce the other enantiomer if our structure is indeed a chiral YSR molecule. The reduced +α shapes are mirrored in the triangle pointing to the left (Figure 2h–j) compared to those of the triangle pointing to the right (Figure 2b–d), revealing the opposite chirality and that the triangles are indeed enantiomers.

Simulations of trimer states within the tight-binding model are shown in Figure 2e,k. We have selected the equal-weight linear combination of the model monomer wave functions for both triangular arrangements. Our simulated wave functions display a structure with reduced symmetry around each atom, essentially highlighting two sides of an equilateral triangle. The orientation of these sides obeys the 3-fold rotational symmetry of the trimer structure. At the same time, the rotational sense inverts from one arrangement to the other. Although the detailed shapes differ from experiment, the model correctly reproduces the observed chirality. We note that the phenomenological model also captures the chirality pattern (see SI, Note 3). Translating this phenomenological model to the sketch of the +α shape delivers a simple picture of the reduced +α shapes as shown in SI Figure S5e,j. To bring out the importance of the adsorption geometry for the appearance of chirality, we also investigate an equilateral triangle with an edge length of three lattice spacings along the [11̅00] direction (i.e., along the atomic Se rows). The corresponding experimental data and the modeled d*I*/d*V* maps are shown in the SI Note 6. In this configuration, *D*_3_ is restored and no chirality is observed.

The chiral patterns of the YSR states are a direct consequence of the broken symmetries of the adatom structure by the underlying surface. An iso-structural chiral molecule does not exist in the gas phase, where chirality cannot occur in planar molecules, but requires three-dimensional structures. The simplest chiral molecule exhibits a single chiral center, e.g., a C atom with different substituents. In such cases, the enantiomers can be labeled according to the Cahn-Ingold-Prelog convention. We also cannot determine a winding direction as there is no helical axis due to the two-dimensional nature. Our realization on the substrate does not allow labeling the enantiomers according to any nomenclature used in gas-phase chemistry. Thus, we assign a sense of rotation to distinguish the shapes.

### Engineering YSR Wave Functions in Larger Fe Structures

**Figure 3:**
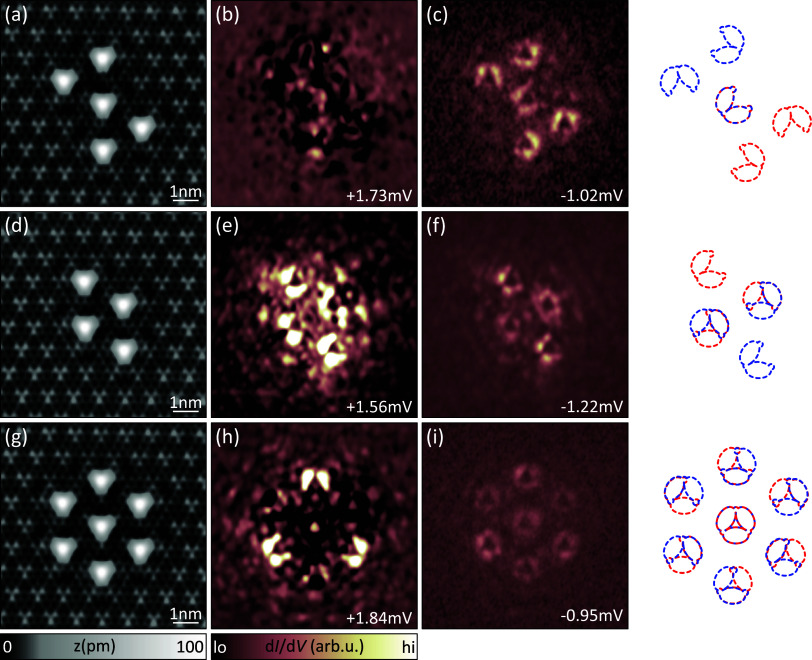
Larger adatom structures. (a) Topography of five Fe atoms arranged in a bowtie pattern. (b, c) Exemplary d*I*/d*V* map of a +α resonance of the bowtie structure and its thermal replica (multiplied by a factor of 10). (d) Topographic image of four Fe atoms arranged into a rhombus. (e, f) d*I*/d*V* map of the rhombus structure at the energies of a +α resonance and its thermal replica. (g) Topography of seven Fe atoms arranged in a hexagon with an additional atom at its center. (h, i) d*I*/d*V* maps of a +α resonance of a hexagon structure and its thermal replica (multiplied by a factor of 10 to compensate for the reduced intensity of thermal replica). Next to the d*I*/d*V* maps we depict overlaid schematics of the reduced +α shapes to match the adatom structures. The different enantiomeres are indicated by different colors (blue and red). Δ_tip_ = 1.41 mV; set points: (a, d, g) 10 mV, 50 pA; rest 5 mV, 700 pA; all: *V*_rms_ = 15 μV.

Next, we exemplify the potential of wave function engineering in larger structures. To this end, we deliberately tailor structures with symmetries which suppress or exhibit chiral patterns. We start by combining two corner-sharing triangles of opposite chiralities. The resulting “bow-tie” structure is shown in Figure 3a. We only show maps of one +α resonance, which features the same details as those shown for the chiral triangular YSR molecules. The d*I*/d*V* map of the original resonance (Figure 3b) is strongly influenced by negative differential conductance as described above. The thermal replica shown in Figure 3c, however, facilitates clear identification of the same patterns observed in Figure 2c,g. The bow-tie molecule has *C*_*s*_ symmetry, where the mirror plane coincides with the mirror plane of the reduced +α shape of the shared atom for each enantiomer. Therefore, the chiral pattern appears just like the one of two triangles of opposite chirality. This behavior is further visualized by a schematic of the bow-tie structure, where the enantiomers are distinguished by color as depicted next to the corresponding d*I*/d*V* maps.

We can alternatively combine two triangles of opposite chirality resulting in a “rhombus” as shown in Figure 3d. Here, two atoms are shared between the triangles and just like the bow tie, the resulting structure has *C*_s_ symmetry. d*I*/d*V* maps of a +α-like resonance as well as its thermal replica are shown in Figure 3e,f, respectively. We only observe clear reduced +α shapes on the nonshared atoms of the structure. However, no two atoms within a chiral structure share a mirror plane. Therefore, the mirror plane of the rhombus cannot coincide with a mirror plane of both atoms that lie on it, as visualized by the schematic depicted next to the maps in Figure 2e,f. As a consequence, the pattern observed on the shared atoms deviates from the reduced +α shape.

We finally reintroduce *D*_3_ symmetry (which we so far only observed in the monomer), by assembling six Fe atoms in an equilateral hexagon and adding a seventh atom in the center. The resulting structure is depicted in Figure 3g. This hexagon could be reduced to any of the previously discussed structures solely by removing atoms. The d*I*/d*V* maps of a +α like resonance and its thermal replica are shown in Figure 3h,i, respectively. When looking at chiral triangles within the hexagon all atoms are shared between triangles. Still the reduced +α shape can be identified in d*I*/d*V* maps. We can therefore trace the states of our YSR molecule back to its parent state in the monomer.

## Conclusions

In conclusion, we showed that crystalline substrates can be used for wave function engineering of adsorbed adatom structures. This opportunity is most relevant when the wave functions originating from hybridization of the adatom states remain unperturbed from bulk states. YSR states are an ideal system as they are protected by the superconducting energy gap. Interestingly, diatomic YSR molecules can break symmetries which cannot be broken in the gas phase. As a result, we can design complex wave function symmetries as in chiral molecules consisting of planar arrangements of one adatom species only.

Our work focused on the design of intricate wave function symmetries. Triangular magnetic adatom structures on superconductors may be even more interesting when including their spin degree of freedom.^35^ Depending on the sign of the exchange interaction, the triangular structures may be ferromagnetic or frustrated. These structures offer rich opportunities for the design of chiral states with complex spin textures, eventually serving as key elements of topologically protected states. In general, broken symmetries, such as broken time-reversal or particle-hole symmetry, can crucially affect transport properties through superconducting junctions leading to nonreciprocal behavior.^36−39^ The influence of chiral structures on transport remains still to be investigated.

## Methods

We achieve a clean and flat 2*H*-NbSe_2_ surface by carbon- or scotch-tape cleaving under ultrahigh vacuum conditions. Fe atoms are deposited directly into the STM at temperatures below 9 K. The as-deposited atoms are found in two distinct adsorption sites as described elsewhere.^25^ We use superconducting Nb tips to increase the energy resolution beyond the Fermi–Dirac limit. As a consequence, all features in differential conductance (d*I*/d*V*) spectra are shifted by the excitation gap of the tip. Note that the data presented here were recorded using different tips with superconducting gaps of approximately 1.55, 1.44, and 1.41 mV (details of the tip preparation can be found in the SI Note 5). The superconducting gap of the tip is indicated by shaded areas in all spectra.

## Data Availability

The original experimental data and the code for the tight-binding model are available at 10.5281/zenodo.13763528.

## Supporting Information Available

Tight-binding model; Phenomenological model; Numerical results for monomer, dimers, and trimers; Details on adsorption site determination; Additional d*I*/d*V* spectra and maps on monomer, dimers, trimers, and larger adatom structures (PDF)
