# Supplementary material for: Wave Function Engineering on Superconducting Substrates: Chiral Yu-Shiba-Rusinov Molecules
Source: ACS Nano. 2024 Oct 25;18(44):30798–804. doi: 10.1021/acsnano.4c10998 (PMC11544926; doi:10.1021/acsnano.4c10998)
Supplement: Supplementary file 1 — nn4c10998_si_001.pdf [file nn4c10998_si_001.pdf]

Supporting Information
Wave-Function Engineering on Superconducting Substrates: Chiral Yu-Shiba-Rusinov Molecules

Lisa M. Rütten,[†] Harald Schmid,[‡] Eva Liebhaber,[†] Giada Franceschi,[†] Ali Yazdani,[†] Gaël Reecht,[†] Kai Rossnagel,[¶,§] Felix von Oppen,[‡] and Katharina J. Franke[*,†]

†*Fachbereich Physik, Freie Universität Berlin, 14195 Berlin, Germany*

‡*Dahlem Center for Complex Quantum Systems and Fachbereich Physik, Freie Universität Berlin, 14195 Berlin, Germany*

¶*Institut für Experimentelle und Angewandte Physik, Christian-Albrechts-Universität zu Kiel, 24098 Kiel, Germany*

§*Ruprecht Haensel Laboratory, Deutsches Elektronen-Synchrotron DESY, 22607 Hamburg, Germany*

E-mail: franke@physik.fu-berlin.de

Supporting Information Note 1: Tight-binding model

We employ an effective tight-binding model to simulate the behavior of YSR molecules on $2H$-$NbSe_2$. We focus on a model for the top layer neglecting the other weakly coupled layers. The top layer is itself a trilayer consisting of a central layer of Nb atoms situated between two layers of Se atoms. Both Nb and Se atoms form triangular sublattices, with the Se sublattice shifted by half a lattice vector relative to the Nb atoms. The trilayer has threefold

rotation symmetry, three mirror symmetries within the plane as well as a horizontal mirror symmetry with respect to the Nb layer (D_{3h} symmetry group).

The Fermi surface consists of pockets at the K-points and around the Γ-point, predominantly deriving from Nb d-orbitals. The experimental maps of the YSR monomers show oscillations of wavelength $\lambda_F = 1\,\mathrm{nm}$, compatible with the Fermi momentum $k_F = 5.34\,\mathrm{nm}^{-1}$ of the K-pockets. This observation suggests that focusing solely on the K-pockets in our model is sufficient to capture the experimental maps. Some tight-binding models of $NbSe_2$ in the literature[1–4] include only Nb orbitals, while neglecting the Se orbitals. The experimental adsorption geometry, however, suggests that exchange coupling to the Se atoms is stronger than to the Nb atoms, and may therefore be relevant.

Our minimal low-energy Hamiltonian of the normal substrate contains nearest-neighbor hopping on a hexagonal lattice of Nb and Se atoms with one orbital each,

$$H_0 = -t \sum_{\langle \mathbf{r},\mathbf{r}' \rangle} \sum_{\sigma} (c^\dagger_{\mathbf{r}\sigma} d_{\mathbf{r}'\sigma} + d^\dagger_{\mathbf{r}'\sigma} c_{\mathbf{r}\sigma}) + V_{\mathrm{Nb}} \sum_{\mathbf{r}\sigma} c^\dagger_{\mathbf{r}\sigma} c_{\mathbf{r}\sigma} + V_{\mathrm{Se}} \sum_{\mathbf{r}'\sigma} d^\dagger_{\mathbf{r}'\sigma} d_{\mathbf{r}'\sigma} \tag{S1}$$

Here, $c^\dagger_{\mathbf{r}}$ ($d^\dagger_{\mathbf{r}'}$) adds an electron with spin σ to site $\mathbf{r}$ ($\mathbf{r}'$) of the Nb (Se) sublattice, t is the hopping amplitude and V_{Nb}, V_{Se} are the sublattice potentials. The dispersion relation is

$$\epsilon(\mathbf{k}) = \frac{V_{\mathrm{Nb}} + V_{\mathrm{Se}}}{2} \pm \frac{1}{2} \sqrt{(V_{\mathrm{Se}} - V_{\mathrm{Nb}})^2 + 4t^2 \left(3 + 2\cos(k_y a) + 4\cos\left(\frac{k_y a}{2}\right)\cos\left(\frac{\sqrt{3}k_x a}{2}\right)\right)} \tag{S2}$$

with lattice constant $a = 0.344\,\mathrm{nm}$. For $V_{\mathrm{Nb}} = V_{\mathrm{Se}} = 0$, this expression is the low-energy description of graphene with linear dispersion at the K-points. For $0 < V_{\mathrm{Nb}} \ll V_{\mathrm{Se}}$, the sublattice potential opens a gap and the Fermi energy (taken at zero energy) is shifted into the lower band to recover the K-pockets characteristic of $NbSe_2$ with strong Nb character. The pockets remain disconnected as long as the shift remains small compared to the bandwidth (for $V_{\mathrm{Se}} V_{\mathrm{Nb}} < t^2$ precisely). Choosing $t = 1\,\mathrm{eV}$ as the unit of energy, we set $V_{\mathrm{Se}}/t = 4$ and

$V_{\mathrm{Nb}}/t = 0.22$ to match the values of the Fermi momentum $k_F a = 1.84$ from the experiment and the Fermi velocity $\hbar v_F/a$ =0.78 eV from Ref. 5. The Fermi surface obtained within our model is depicted in Fig. S1. The model fails to reproduce the Γ-pockets. It also does not reproduce the weak corrugation of the K-pockets along the c axis, which reflects the weak interlayer coupling. We also neglect spin-orbit coupling[6] and the presence of the charge-density wave.[7] While the former splits the Fermi pockets at the K-points, leading to slight differences in k_F for different spins, the latter respects the symmetry of the adsorption geometry. Neither effect is expected to impact the symmetry of local YSR wave functions.

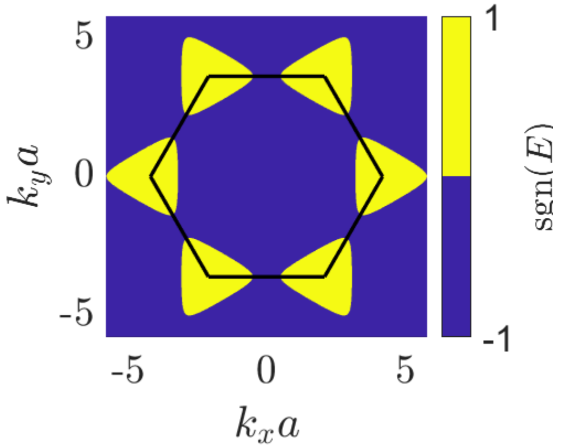

Figure S1: Fermi surface of $NbSe_2$ as obtained from our model.

The superconducting state of $NbSe_2$ exhibits at least two distinct superconducting gaps, indicating either anisotropic or multiband pairing.[8] However, the anisotropic nature of the gap affects the YSR wave function on scales of the coherence length $\xi \simeq 240\,\mathrm{nm}$ which greatly exceeds the size of YSR molecules studied in our experiment ($d < 3\,\mathrm{nm}$). Hence, we include superconductivity as conventional BCS s-wave pairing of equal strength $\Delta/t = 0.02$ for both Nb and Se atoms, with $\xi \simeq 20\,\mathrm{nm} \gg d$. For numerical reasons, we choose $\Delta = 20\,\mathrm{meV}$, which is an order of magnitude larger than the experimental value of $\Delta_{\mathrm{exp}} \simeq 1\,\mathrm{meV}$. This facilitates the use of smaller lattices with 160×160 sites, and should be adequate for modeling the wave function of YSR molecules.

In our model, we treat the magnetic adatoms as classical magnetic moments. The

adatoms induce exchange and potential scattering of conduction electrons. It is crucial to account for the local adsorption geometry in the experiment, where the adatoms stabilize in a D_3-symmetric configuration at the center of a lattice hexagon, as shown in Fig. S2(a). Exchange and potential scattering processes of conduction electrons via d orbitals of the magnetic adatom effectively couple with and between all the neighboring sites. It is essential to retain the coupling between different sites so that one obtains only a single YSR subgap state at positive energies per adatom. (In the absence of the coupling between sites, one would obtain one YSR state per coupled site.) Our model allows for coupling to both Se and Nb atoms,

$$
\begin{aligned}
H_I =& \sum_{\sigma\sigma'} \sum_{\mathbf{e}_i \mathbf{e}_j} c^\dagger_{\mathbf{e}_i\sigma} \left(\frac{K_{\text{Nb}}}{3} \delta_{\sigma\sigma'} - \frac{J_{\text{Nb}}}{3} \sigma^z_{\sigma\sigma'} \right) c_{\mathbf{e}_j\sigma'} + \sum_{\sigma\sigma'} \sum_{\mathbf{e}'_i \mathbf{e}'_j} d^\dagger_{\mathbf{e}'_i\sigma} \left(\frac{K_{\text{Se}}}{3} \delta_{\sigma\sigma'} - \frac{J_{\text{Se}}}{3} \sigma^z_{\sigma\sigma'} \right) d_{\mathbf{e}'_j\sigma'} \\
&+ \sum_{\sigma\sigma'} \left(\sum_{\mathbf{e}_i \mathbf{e}'_j} c^\dagger_{\mathbf{e}_i\sigma} \left(\frac{K_{\text{Nb,Se}}}{3} \delta_{\sigma\sigma'} - \frac{J_{\text{Nb,Se}}}{3} \sigma^z_{\sigma\sigma'} \right) d_{\mathbf{e}'_j\sigma'} + \text{h.c.} \right), \qquad \text{(S3)}
\end{aligned}
$$

where the vectors $\mathbf{e}_i$ ($\mathbf{e}'_i$) with $i = 1, 2, 3$ locally connect the three neighboring Nb (Se) atoms to the impurity, J_{Nb} (J_{Se}) is the intra-atom exchange scattering between Nb (Se) atoms, $J_{\text{Nb,Se}} = \sqrt{J_{\text{Nb}} J_{\text{Se}}}$ the inter-atom exchange scattering from Nb to Se (and back) and K_{Nb}, K_{Se} and $K_{\text{Nb,Se}} = \sqrt{K_{\text{Nb}} K_{\text{Se}}}$ the corresponding potential scattering amplitudes. We choose $J_{\text{Se}}/t = 0.16$, $J_{\text{Nb}}/t = 0.1$, $K_{\text{Nb}}/t = 0.05$ and $K_{\text{Se}}/t = 0$. This choice reflects that the adatoms are closer to the upper Se layer than to the Nb layer. Larger adatom structures such as those probed in experiment are treated accordingly.

Supporting Information Note 2: Phenomenological model

We qualitatively compare our tight-binding simulations to a phenomenological model in which we assume that the YSR wave function can be viewed as superpositions of circular waves emanating from the three Nb neighbors of the adatom. The lattice structure enters

only through the locations from which the circular waves emanate, but is otherwise effectively neglected. In particular, the phenomenological model neglects the long-range behavior of YSR states associated with the Fermi-surface geometry. Nevertheless, the phenomenological model is helpful in interpreting the symmetries of the measured YSR states as well as the YSR wave functions obtained from the tight-binding model in the vicinity of the impurity. In our local picture, the electron (hole) wave function ϕ_+ (ϕ_-) emanating from the Nb atom at $\mathbf{r}_i$ has the form of a YSR state in 2D with s-wave symmetry,[4]

$$\phi_{i,\pm}(|\mathbf{r}-\mathbf{r}_i|) = \frac{1}{\sqrt{N\pi k_F|\mathbf{r}-\mathbf{r}_i|}} \sin\Big(k_F|\mathbf{r}-\mathbf{r}_i| - \frac{\pi}{4} + \delta^{\pm}\Big) e^{-\sin\left(\delta^+-\delta^-\right)|\mathbf{r}-\mathbf{r}_i|/\xi}, \tag{S4}$$

with the scattering phase shifts $\delta^{\pm}$ determined by potential scattering, superconducting coherence length ξ and normalization constant N. The monomer wave function is taken to be a symmetric superposition of the circular waves emanating from the three Nb scatterers located on the vertices of an equilateral triangle,

$$\psi_{M,\pm}(\mathbf{r}) = \frac{1}{\sqrt{3}} \sum_{i=1}^{3} \phi_{i,\pm}(|\mathbf{r}-\mathbf{r}_i|). \tag{S5}$$

Dimer and trimer wave functions will be constructed by further linear combinations of monomer wave functions in the experimental configurations, assuming equal overlaps of the monomer states.

Supporting Information Note 3: Numerical results

Our tight-binding model provides qualitative understanding of the experimental dI/dV maps of YSR molecules. While symmetry features are reproduced, our simulations do not aim at quantitative agreement with the experimental data in view of the simplicity of the model.

Monomer

In Fig. 1(h) of the main text we show the electronic part of the simulated monomer wave functions ($|\psi|^2$) with mid-gap energy. The wave function reflects the D_3-symmetry of the local adsorption geometry. Notably, the maxima are directed towards the neighboring Se atoms of the Fe adatom, consistent with experiment and a density functional theory calculation.[9] This feature originates from exchange coupling to the Nb atoms, and is insensitive to moderate variations in $J_{\mathrm{Nb}}/J_{\mathrm{Se}}$ given the strong Nb character of the Fermi surface. We also find that potential scattering tends to align the maxima with the Se neighbors. The direction of the wave function may be further stabilized by the charge-density wave maximum. Far from the adatom, long-range oscillations mirror the hexagonal shape of the Fermi surface. The detailed structure of the monomer wave function in the vicinity of the impurity is largely determined by the d-orbital physics of the Fe adatom, limiting our calculation to qualitative symmetry features.

The phenomenological model gives a monomer wave function (Fig. S2b), which is qualitatively consistent with the tight-binding calculation in the vicinity of the impurity. Further away from the impurity, our ansatz shows spherically symmetric oscillations with k_F, in contrast to the hexagonal oscillation pattern observed in the tight-binding simulation and in experiment.

Dimers

Figure S2c (and Fig. 1g of the main text) illustrate the lattice positions of the adatoms in the dimer configuration probed in the experiment. The local adsorption geometry of the adatoms breaks the mirror symmetry with respect to a plane perpendicular to the dimer axis. Our simulations assume the same coupling for the two adatoms (Eq. (S3)).

In Fig. 1i, j of the main text, we show the local density of states of the simulated dimer YSR wave function (tight-binding model). The simulated wave functions reflect the broken mirror symmetry in their intensity pattern on the different adatoms, qualitatively agreeing

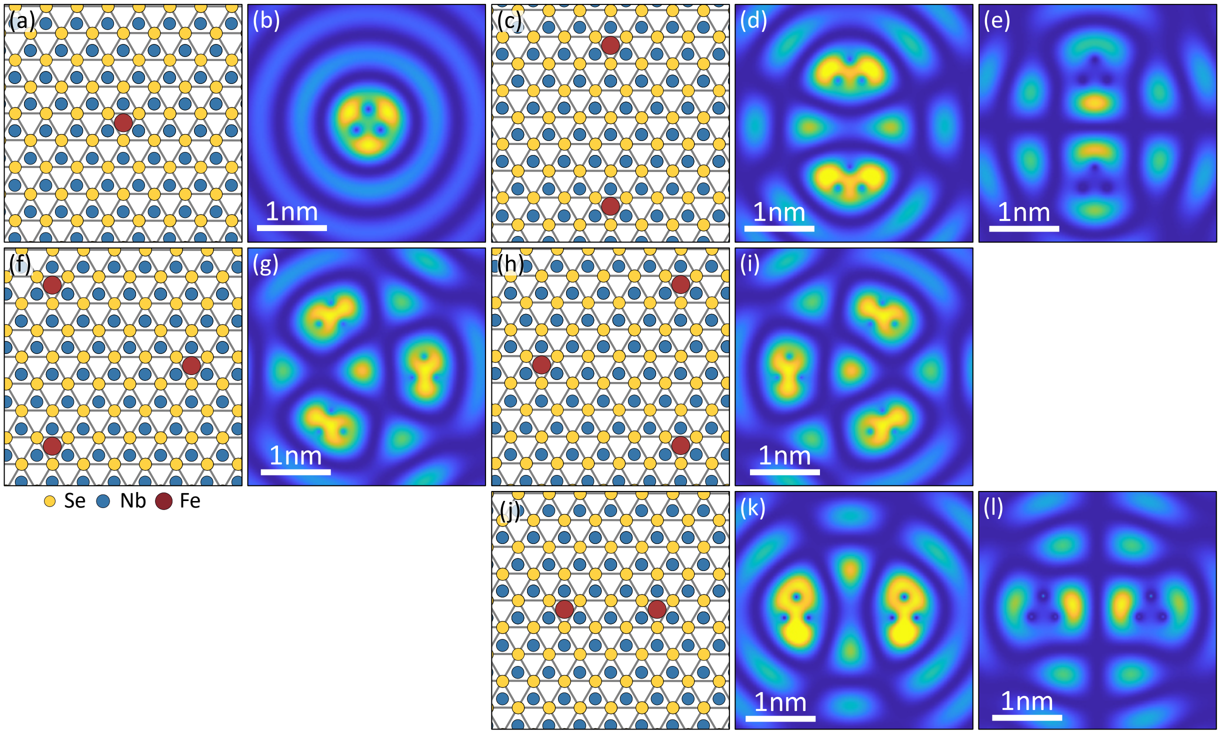

Figure S2: (a), (c), (f), (h), (j) Adsorption geometries of the monomer (a), dimer along the $[11\bar{2}0]$ direction (c), trimers in configuration A (f) and B (h) formed by adding a third atom on either side of the dimer, and the dimer along the $[1\bar{1}00]$ direction (j). (b), (d), (e), (g), (i), (k), (l) Wave functions obtained from the phenomenological model. Shown is the electronic part of the monomer wave function ($|\psi|^2$) (b), symmetric (d), (k) and anti-symmetric (e), (l) dimer configurations for the dimer along the $[11\bar{2}0]$ (d), (e) and along the $[1\bar{1}00]$ (k), (l) direction, and trimer enantiomers A (g) and B (i).

with the experiment. The intensity patterns originate from the far-field wave fronts from one adatom interfering with the near-field wave function close to the other adatom. We have confirmed that the dimer wave functions are symmetric and antisymmetric linear combinations of the monomer wave functions. Interestingly, the antisymmetric wave function lacks a nodal plane along the broken mirror axis since the monomer wave function is not mirror-symmetric with respect to its horizontal axis.

Comparing the tight-binding simulations with the phenomenological model (Fig. S2b, d, e, g, i, k, l) on a qualitative level, we find that both break the mirror symmetry. However, the antisymmetric wave function of the phenomenological model (Fig. S2e) displays a nodal line, in contrast to the tight-binding model (main text Fig. 1). This inconsistency arises because the phenomenological monomer wave function already exhibits approximately isotropic long-range behavior at the dimer center. In contrast, the monomer wave functions of the tight-binding model are still dominated by the threefold-symmetric short-range behavior at the dimer center, suppressing the nodal plane. Hence, the absence of the nodal plane as observed experimentally is only confirmed in the tight-binding simulation.

We also simulate dimers in a mirror-symmetric configuration (cf. Fig. S2 k, l and Fig. S4i, j) placed at a distance $d' = 3a = 1.03\,\mathrm{nm}$, as investigated experimentally in Ref. 10. The wave function of both symmetric and anti-symmetric YSR states (Fig. S4i, j, respectively) reflect the mirror symmetry of the adatom configuration, and exhibit a nodal plane for the anti-symmetric YSR state. The phenomenological model (Fig. S2k, l) yields wave functions that share these properties.

Trimers

In the trimer, a third adatom is added to the dimer configuration, such that the three adatoms form an equilateral triangle with edge length d, see Fig. S2f, h. One can place the third adatom next to the dimer in two inequivalent ways (dubbed trimer configuration A and B) related by a mirror operation, with the original dimer axis acting as the mirror

plane. Due to the adsorption geometry, the trimer has three-fold rotation symmetry, but the mirror symmetry with respect to a plane perpendicular to the edges of the adatom triangle is broken.

In our tight-binding simulation, we find three mid-gap YSR trimer states of which two are degenerate. This finding is consistent with a linear combination of the YSR monomer wave function, assuming equal hybridization between the monomer wave functions and time-reversal symmetry such that the overlap integral is real. With this ansatz, we get the trimer wave functions

$$\begin{aligned}\psi_{T,0}(\mathbf{r}) &= \sqrt{\frac{1}{3}}\Big(\psi_M(\mathbf{r}-\mathbf{R}_1)+\psi_M(\mathbf{r}-\mathbf{R}_2)+\psi_M(\mathbf{r}-\mathbf{R}_3)\Big),\\ \psi_{T,+/-}(\mathbf{r}) &= \sqrt{\frac{1}{2}}\Big(\psi_M\left(\mathbf{R}-\mathbf{R}_1\right)-\psi_M(\mathbf{r}-\mathbf{R}_{2/3})\Big),\end{aligned} \tag{S6}$$

where $\mathbf{R}_i$ are the three vectors from the trimer center to the impurities. The wave functions $\psi_{T,+/-}$ are degenerate (so any linear combination is also possible). In the following, we focus on $\psi_{T,0}$ which is shown in Fig. 2e of the main text (configuration A).

The trimer wave function in Fig. 2e of the main text again has trilateral symmetry but no reflection symmetry. Similar to the experiment, the wave function pattern in the vicinity of each adatom describes a v-shape, opening to one of the other adatoms in a clockwise manner (positive chirality). Switching from configuration A to B, the wave function pattern is also mirrored (Fig. 2k of the main text). The opening of the v-shapes now is counterclockwise (negative chirality).

The chirality of the wave function pattern can be readily understood from the linear combination of monomer states. Our wave function ansatz $\psi_{T,0}(\mathbf{r})$ is invariant under cyclic exchange of impurity vectors $\mathbf{R}_i$ reflecting the threefold rotation symmetry. However, changing the trimer configuration from A to B sends $\mathbf{R}_i \to -\mathbf{R}_i$ in $\psi_{T,0}(\mathbf{r})$. Thus, the rotational direction of the wave function pattern is reversed. The tight-binding simulation (Fig. 2e, k in main text) and the phenomenological model (Fig. S2g, i) make consistent predictions.

Supporting Information Note 4: Adsorption sites and incommensurate CDW

Single Fe atoms on the clean $NbSe_2$ surface adsorb in two distinct sites. They can be distinguished by their different apparent height and shape and identified as sitting in two distinct hollow sites of the terminating Se layer (one with a Nb atom underneath - metal site (MS), the other one without one - hollow site (HS)) as shown in Ref. 11, and in the supplementary material of Ref. 10. Furthermore, the YSR states of Fe atoms adsorbed in hollow sites vary significantly for different adsorption sites with respect to the CDW. Details can be found in Refs. 10 and 11. Here, we only investigate Fe atoms that sit in hollow sites on maxima of the CDW (same as atom I in Ref. 11, which also constituted the start of the chain in Ref. 10).

Supporting Information Note 5: Superconducting Nb tips

All measurements were performed with superconducting Nb tips. The tips were prepared by indenting bulk wire tips (W for the data on chiral triangles, NbTi for all other data) into a superconducting Nb sample until a sharp, stable apex and an (almost) full bulk-like superconducting gap ($\Delta \approx 1.55\,\mathrm{meV}$) was obtained. Small tip indentations were performed on the $NbSe_2$ sample to obtain a tip apex suitable for controlled lateral manipulation of the Fe atoms. Sometimes, the superconducting gap of the tip became smaller during sample exchange or small tip formings on $NbSe_2$. Therefore, the tips used to record different data sets exhibit different superconducting gap sizes.

Because we probe the energetically sharp YSR resonances with the sharp coherence peaks of the superconducting tip, we frequently observe negative differential conductance (NDC). In $\mathrm{d}I/\mathrm{d}V$ maps, NDC manifests as black patches, such that we do not resolve the actual

spatial structure of a resonance. To disentangle the contributions of a resonance from its negative differential conductance, we exploit the fact that we observe thermal replica at our measurement temperature of those resonances that are deep inside the superconducting gap. These thermal peaks suffer much less from NDC as they are of lower intensity. In brief, the replica originate from thermally excited quasiparticle tunneling. Using a superconducting tip with the gap size Δ_{tip}, a YSR state at energy ϵ is probed at an energy $eV = \pm(\Delta_{\mathrm{tip}} + \epsilon)$. At finite temperature, thermal excitations lead to a partial occupation of originally unoccupied states (and vice versa). Hence, tunneling is also possible at energies $eV = \mp(\Delta_{\mathrm{tip}} - \epsilon)$, i.e., inside the gray area marked in the spectra. The corresponding peak intensities are given by the thermal excitation probability and, thus, they are much less intense than the original peaks. More details on the tunneling processes involving sub-gap states in superconductors can be found in Ref. 12.

The atoms could be moved and positioned with high precision by laterally approaching them with the STM tip in constant-current mode at set points below 10 nA at bias voltages around 5 mV (the exact values depend on the tip apex). We then slowly move the tip using the follow-me option in the Nanonis software and drag the atom across the surface. Each jump of the atom to a new adsorption site can be observed in the real-time current and tip-height charts. To release the atom, we change the set point to normal scanning parameters.

Supporting Information Note 6: Additional data

Here, we present additional data recorded on the structures discussed in the main manuscript as well as some complementary data on a dimer and a trimer arranged along the $[1\bar{1}00]$ direction.

Monomer and dimers

As mentioned in the main text, the $+\alpha_2$ map of the dimer exhibits characteristics of a monomer's $+\beta$ resonance alongside the characteristic $+\alpha$ shape. Here we explain this behavior and its origin in more detail and discuss the assignment of resonances as "$+\alpha$".

In Fig. S3c-f we show $\mathrm{d}I/\mathrm{d}V$ maps of the monomer's α and β resonances at both bias polarities ($+\alpha$ reproduced from main text). Note that both the α and the β state display distinctly different patterns and also the electron-like and the hole-like component of the same state are very different. These differences enable us to track the $+\alpha$ resonance even when it crosses the Fermi level. The characteristic $+\alpha$ and $+\beta$ shapes are overlayed as a guide to the eye in Fig. S3d and f, respectively. The $+\beta$ resonance has the same overall triangular characteristics as the $+\alpha$ shape but the intensity is shifted from the edges (lobes of the $+\alpha$ resonance) to the corners, where we observe bright circles. Generally, YSR wave functions inherit their spatial characteristics close to the adatom position from the d-level that hosts the impurity spin. Therefore, YSR states originating from spins in different d-levels usually exhibit different wave-function patterns. Due to the complexity of our substrate, we cannot assign our YSR states to distinct d-levels, but still observe different patterns for different YSR states.

We further reproduce the dimer's $\mathrm{d}I/\mathrm{d}V$ maps shown in the main text and show their opposite-polarity counter parts in Fig. S3k-n. We overlay the characteristic $+\alpha$ and $+\beta$ shape in Fig. S3n, which are labeled $+\alpha_2, +\beta_1$ here. Just as the $+\alpha$ shape, the $+\beta$ shape is reduced and only exhibits one mirror plane in the dimer. Finding a signature of the β state concomitant to the α state shows that the hybridization-split states become almost degenerate.

In Fig. S3h we additionally show a false-color plot of spectra recorded along the dimer axis ($[11\bar{2}0]$ direction). The absence of a mirror plane between the atoms for any resonance is clearly visible in this plot. The inset shows a topography with an arrow indicating where the line of spectra was taken. In agreement with the $\mathrm{d}I/\mathrm{d}V$ maps, all resonances that are

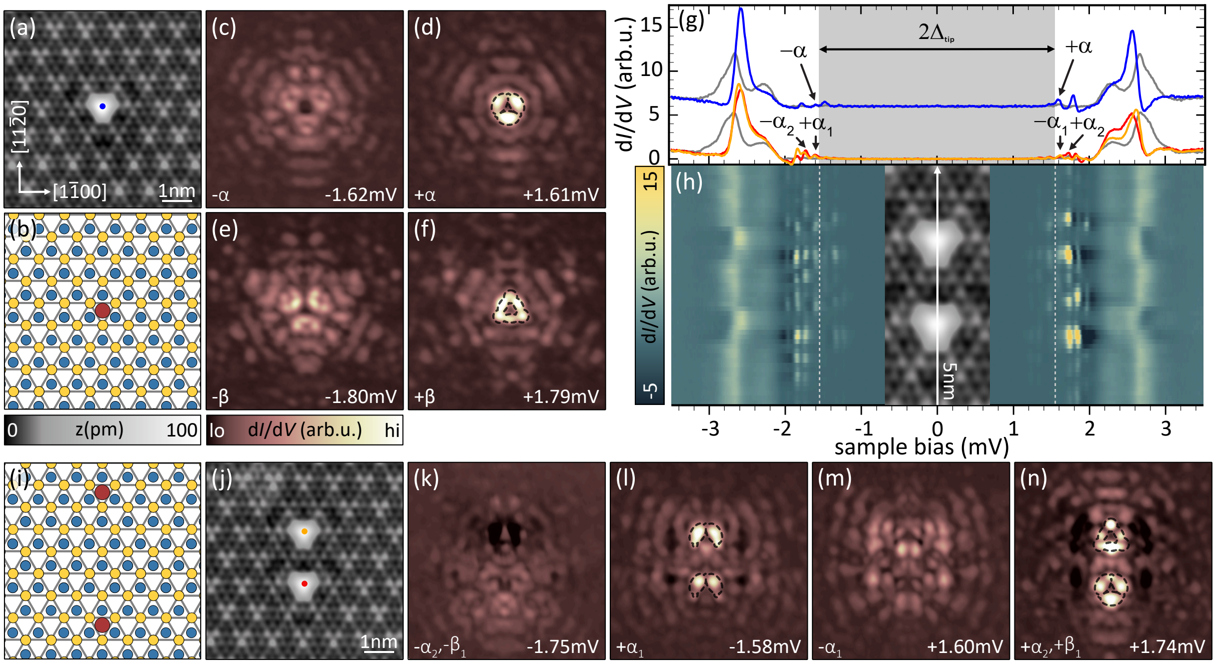

Figure S3: (a), (j) Topographic images of monomer and dimer configurations reproduced from the main text. (b), (i) Schematic adsorption geometries of the monomer (b) and the dimer along the [11$\bar{2}$0] direction (i). (c-f) dI/dV maps of the α and β resonances of the monomer recorded at both bias polarities. (g) Spectra recorded at the positions indicated by the colored dots in the topographic images. (h) False-color plot of spectra recorded along the dimer axis (arrow depicted in the inset). (k-n) dI/dV maps of the two α resonances observed on the dimer at both bias polarities. $\Delta_{\mathrm{tip}} = 1.55\,\mathrm{mV}$; set point: (a),(j), inset in (h) 10 mV, 100 pA; (c-f), top row of (g) 5 mV, 250 pA; bottom row of (g), (h), (k-n) 5 mV, 700 pA; all: V_{rms}=15 μV.

found deep inside the gap exhibit maximal intensity next to each atoms center rather than centrally on it. Therefore the resonances appear to have very low intensities in the spectra shown in Fig. S3g, which are the same spectra shown in the main text of this manuscript.

To highlight the symmetry properties of the dimer along $[11\bar{2}0]$, we show data recorded on a dimer arranged along the $[1\bar{1}00]$ direction in Fig. S4 for comparison. As visible in Fig. S4b, both atoms exhibit equivalent spectra as expected for hybridized YSR states in dimers with a mirror plane between the atoms and also observed in previous experiments.[10] Figure S4c-h show differential conductance maps recorded at the energies indicated by vertical dashed lines in Fig. S4b. Note, that there is a mirror plane visible between both atoms in all maps and symmetric and antisymmetric hybrid YSR states can be distinguished by a nodal line along this mirror plane. Figure S4g and h show thermal replica of the maps depicted in e and g, respectively. A detailed analysis and discussion can be found in Ref. 10. We model YSR wave functions of this dimer using the same tight-binding approach discussed in Note 1 and 3 as well as the main text. The resulting intensity distributions for two resonances are shown in Fig. S4i, j. For this dimer the model yields a nodal plane perpendicular to the dimer axis for the antisymmetric combination of the monomer YSR states, which we also observe in the experiment.

Triangles

We repeat the topographic images and $\mathrm{d}I/\mathrm{d}V$ maps of the chiral triangles from the main text in Fig. S5, and add $\mathrm{d}I/\mathrm{d}V$ maps of the same YSR state at negative bias (right panels of Fig. S5b, g). The chirality is less obvious in these maps than in the ones at positive bias voltages but can still be identified for example in form of a windmill-like structure at the center of each map. In the triangles, the reduced $+\alpha$ shapes appear rotated compared to those expected for each dimer forming a side of the triangle. This rotation can be understood in a naive picture, when overlaying the reduced $+\alpha$ shapes expected for each side of the triangle and assuming destructive interference. We show a sketch of this scenario in Fig. S5e,

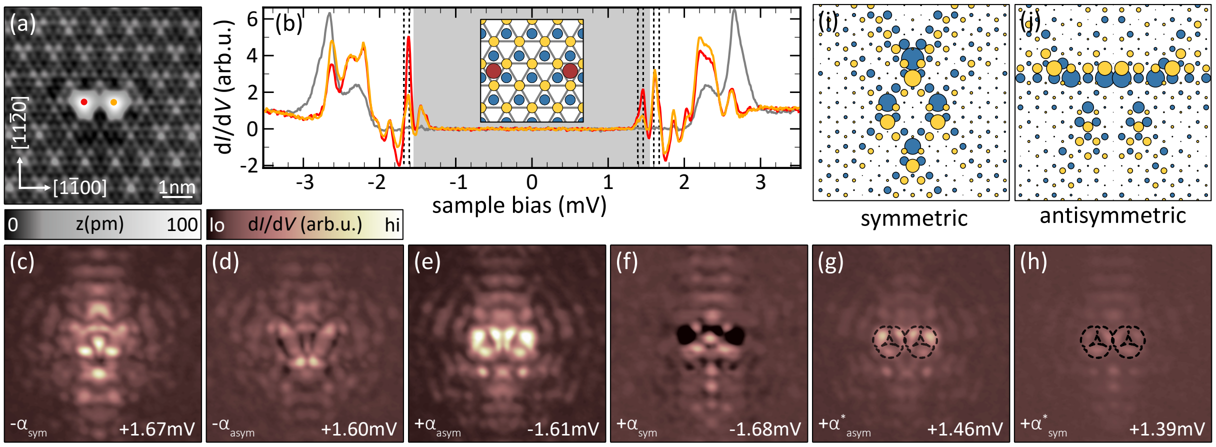

Figure S4: (a) Topographic image of a Fe dimer arranged along the [1$\bar{1}$00] direction at a distance of three lattice spacings (≈ 1 nm). (b) Spectra recorded on both atoms (positions indicated by dots of corresponding color in (a)). (c-f) dI/dV maps of both hybrid α YSR states at both bias polarities. (g, h) dI/dV maps of the thermal replica of the states mapped in (e, f). The energies at which all maps were recorded are indicated by dashed vertical lines in (b). (i, j) Numerical results for this dimer. $\Delta_{\rm tip} = 1.55$ mV; set point: (a) 10 mV, 100 pA; rest 5 mV, 250 pA; all: $V_{\rm rms}$=15 μV.

j where we overlay the reduced $+\alpha$ shapes of three dimers to form triangles. At each corner, one lobe of the corner-forming dimers' reduced $+\alpha$ shape coincides and therefore cancels when assuming destructive interference. The corresponding lobes are shaded in gray in Fig. S5e, j and the remaining lobes match our observations. We further present dI/dV maps of a β-like YSR state of the chiral triangles in Fig. S5c, h. Again the chirality is more obvious in the maps at positive bias, where we also overlay the characteristic $+\beta$ shape (see Fig. S3f, n for monomer and dimer). In the main text we chose to show dI/dV maps of the sharpest YSR resonance, which also exhibits the clearest reduced $+\alpha$ shape and very obvious chirality. Other than for the monomer and dimer, the clearest resonance is not the one lowest in energy for the triangles. For completeness we show maps of the lowest resonances in Fig. S5d, i.

As for the dimer, we also built a triangle with sides along the [1$\bar{1}$00] direction. The corresponding data set is shown in Fig. S6. The atoms within this structure are at closer distance, than the ones in the triangles shown in the main text ($\approx$1 nm instead of $\approx \sqrt{3}$ nm), as visible in the topographic image shown in Fig. S6a. Spectra recorded on each of the atoms are shown in Fig. S6b, where vertical dashed lines indicate energies at which the dI/dV maps

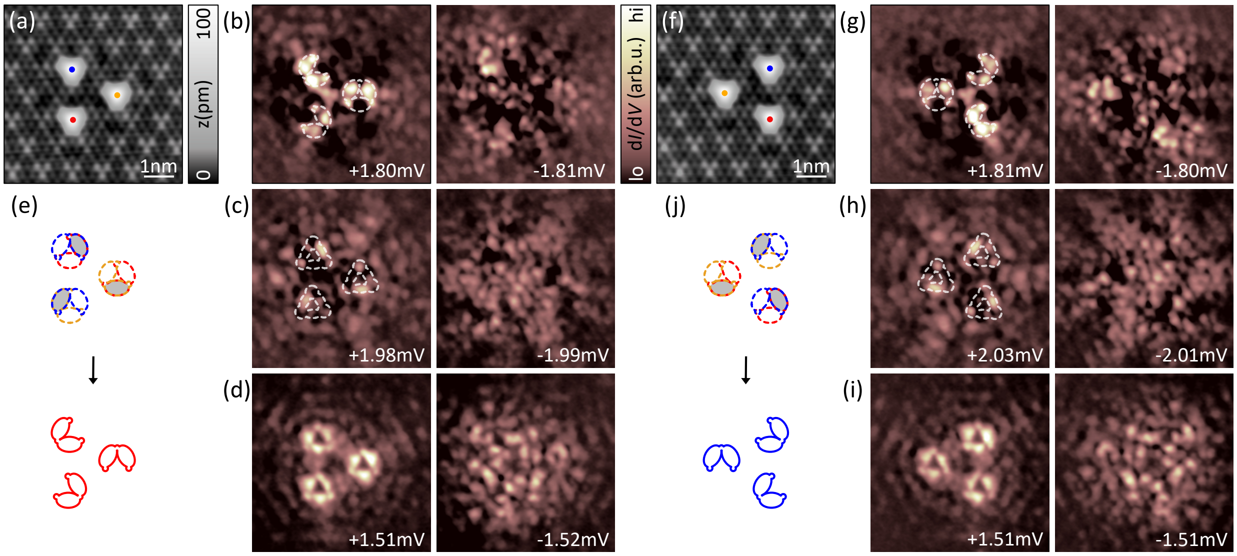

Figure S5: (a, f) Topographic images of both enantiomer triangles reproduced from the main text. (b, g) dI/dV maps of a $+\alpha$-like resonance (left) and the corresponding negative-bias resonance of each enantiomer (left panels reproduced from the main text). (c, h) dI/dV maps at both bias polarities of a β-like YSR state for both enantiomers. (d, i) dI/dV maps at both bias polarities of the lowest-energy YSR state of each enantiomer. (e, j) Overlayed schematics of the reduced $+\alpha$ shapes expected for each side of the triangles. $\Delta_{\mathrm{tip}} = 1.44\,\mathrm{mV}$; set point: (a), (f) 10 mV, 50 pA; all others 5 mV, 750 pA; all: $\mathrm{V_{rms}}$=15 μV.

shown in c and d were recorded. The dI/dV maps do not show any chiral features but C_{3v} symmetry. The modeled wave function captures the over all triangular shape with little intensity at the trinangle's center observed in experiments.

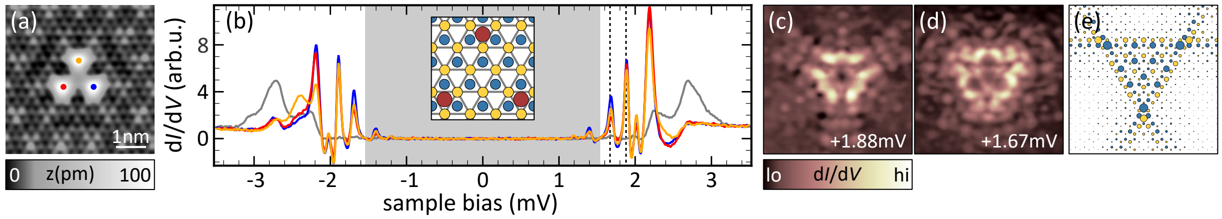

Figure S6: (a) STM topography of three Fe atoms arranged into an equilateral triangle with edges along the $[1\bar{1}00]$ direction. (b) Spectra recorded on each atom of the trimer. (c, d) dI/dV maps of two exemplary YSR states (energies indicated by vertical dashed lines in (b)). $\Delta_{\mathrm{tip}} = 1.55\,\mathrm{mV}$; set point: (a) 10 mV, 100 pA; rest 5 mV, 250 pA; all: $\mathrm{V_{rms}}$=15 μV.

Beyond trimers

Figure S7 shows spectra recorded on each atom of the bow-tie (a), rhombus (b), and hexagon (c) structures along with the same topographic images shown in the main text. The positions, at which each spectrum was recorded, are labeled and color coded in the topographies. All spectra that should be equivalent following symmetry arguments are displayed with the same offset along the y-axis. All of the spectra exhibit several YSR states that overlap within our energy resolution.

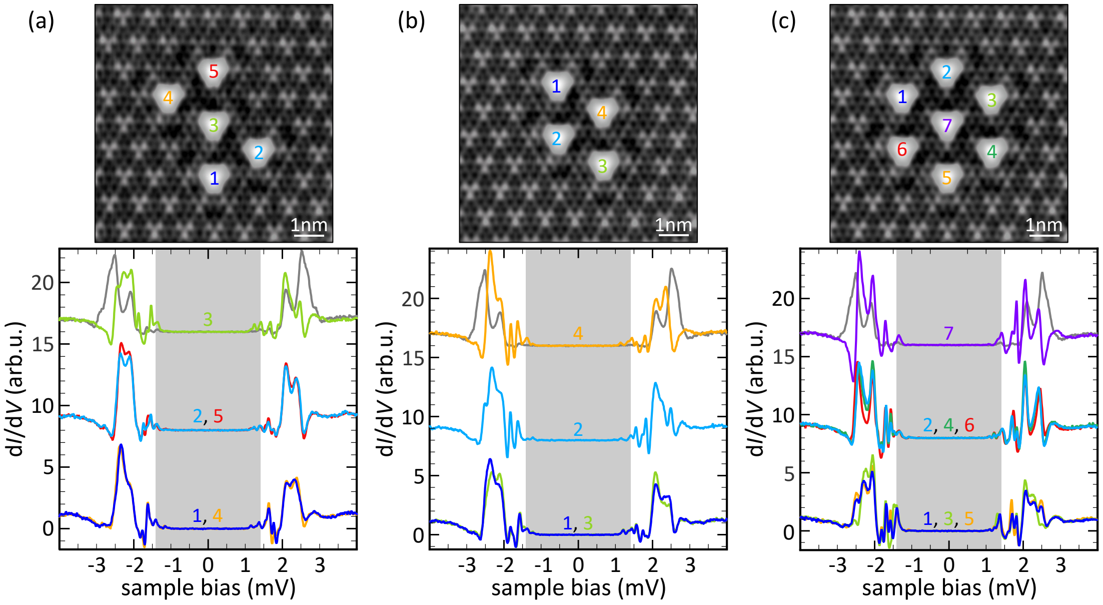

Figure S7: Spectra recorded on all atoms of the bow-tie, rhombus, and hexagon structures with the positions indicated in topographic images above. $\Delta_{tip} = 1.41\,mV$; set point: topographies 10 mV, 50 pA; spectra 5 mV, 700 pA; all: V_{rms}=15 μV.

References

1. Smith, N. V.; Kevan, S. D.; DiSalvo, F. J. Band structures of the layer compounds $1T$-TaS_2 and $2H$-$TaSe_2$ in the presence of commensurate charge-density waves. *J. Phys. Condens. Matter* **1985**, *18*, 3175.

2. Rossnagel, K.; Rotenberg, E.; Koh, H.; Smith, N. V.; Kipp, L. Fermi surface, charge-density-wave gap, and kinks in $2H$-$TaSe_2$. *Phys. Rev. B* **2005**, *72*, 121103.

3. Inosov, D.; Zabolotnyy, V.; Evtushinsky, D.; Kordyuk, A.; Büchner, B.; Follath, R.; Berger, H.; Borisenko, S. Fermi surface nesting in several transition metal dichalcogenides. *New J. Phys.* **2008**, *10*, 125027.

4. Ménard, G. C.; Guissart, S.; Brun, C.; Pons, S.; Stolyarov, V. S.; Debontridder, F.; Leclerc, M. V.; Janod, E.; Cario, L.; Roditchev, D.; Simon, P.; Cren, T. Coherent long-range magnetic bound states in a superconductor. *Nat. Phys.* **2015**, *11*, 1013.

5. Sticlet, D.; Morari, C. Topological superconductivity from magnetic impurities on monolayer $NbSe_2$. *Phys. Rev. B* **2019**, *100*, 075420.

6. Xi, X.; Wang, Z.; Zhao, W.; Park, J.-H.; Law, K. T.; Berger, H.; Forró, L.; Shan, J.; Mak, K. F. Ising pairing in superconducting $NbSe_2$ atomic layers. *Nat. Phys.* **2016**, *12*, 139–143.

7. Borisenko, S. V.; Kordyuk, A. A.; Zabolotnyy, V. B.; Inosov, D. S.; Evtushinsky, D.; Büchner, B.; Yaresko, A. N.; Varykhalov, A.; Follath, R.; Eberhardt, W.; Patthey, L.; Berger, H. Two Energy Gaps and Fermi-Surface "Arcs" in $NbSe_2$. *Phys. Rev. Lett.* **2009**, *102*, 166402.

8. Sanna, A.; Pellegrini, C.; Liebhaber, E.; Rossnagel, K.; Franke, K. J.; Gross, E. K. U. Real-space anisotropy of the superconducting gap in the charge-density wave material $2H$-$NbSe_2$. *npj Quantum Mater.* **2022**, *7*, 1–7.

9. Yang, X.; Yuan, Y.; Peng, Y.; Minamitani, E.; Peng, L.; Xian, J.-J.; Zhang, W.-H.; Fu, Y.-S. Observation of short-range Yu-Shiba-Rusinov states with threefold symmetry in layered superconductor $2H$-$NbSe_2$. *Nanoscale* **2020**, *12*, 8174–8179.

10. Liebhaber, E.; Rütten, L. M.; Reecht, G.; Steiner, J. F.; Rohlf, S.; Rossnagel, K.; von Oppen, F.; Franke, K. J. Quantum spins and hybridization in artificially-constructed chains of magnetic adatoms on a superconductor. *Nat. Commun.* **2022**, *13*, 1–11.

11. Liebhaber, E.; Acero González, S.; Baba, R.; Reecht, G.; Heinrich, B. W.; Rohlf, S.; Rossnagel, K.; von Oppen, F.; Franke, K. J. Yu–Shiba–Rusinov states in the charge-density modulated superconductor $NbSe_2$. *Nano Lett.* **2020**, *20*, 339–344.

12. Ruby, M.; Pientka, F.; Peng, Y.; von Oppen, F.; Heinrich, B. W.; Franke, K. J. Tunneling Processes into Localized Subgap States in Superconductors. *Phys. Rev. Lett.* **2015**, *115*, 087001.
